# cometrics: A New Software Tool for Behavior-analytic Clinicians and Machine Learning Researchers

**DOI:** 10.1007/s40617-023-00817-w

**Published:** 2023-06-08

**Authors:** Walker S. Arce, Seth G. Walker, Morgan L. Hurtz, James E. Gehringer

**Affiliations:** 1 Munroe-Meyer Institute, University of Nebraska Medical Center, 985450 Nebraska Medical Center, Omaha, NE 98198 USA; 2 Electrical and Computer Engineering Department, University of Nebraska-Lincoln, Lincoln, NE 68508 USA

**Keywords:** computerized data collection, Empatica E4, machine learning, observational data, video annotation

## Abstract

Cometrics is a Microsoft Windows compatible clinical tool for the collection and recording of frequency- and duration-based target behaviors, physiological signals, and video data. This software package is designed to record in-vivo observational and physiological data. In addition, we have included features that allow observers to capture video from real-time camera feeds and import saved video for retroactive data collection. By using Microsoft Excel-based spreadsheets, also called keystroke files, assessment and treatment sessions are exported into a single document using the click of a button. Integrated interobserver agreement metrics allow comparisons across primary and reliability observers, with the output exported into a spreadsheet for easy reference. All file system interactions are handled by the user interface, so files and folders are created and managed without manual intervention. This software is available free-of-charge through the Microsoft Store for Windows 10 and 11 and the source code is publicly available on GitHub.

**Supplementary Information:**

The online version contains supplementary material available at 10.1007/s40617-023-00817-w.

Behavior analysts rely on the efficient recording and analysis of observational data to inform clinical decision making and execute research studies. Kostewicz et al. (2016) reviewed data collection procedures across behavior therapy, experimental, and applied behavior-analytic journals, concluding that most publications reported the use of observational data collection in measuring dependent variables of interest. It is reasonable to assume that most clinicians use observational strategies to collect data in applied practice. Unfortunately, many posit that the most accurate methods (i.e., continuous methods) of observational data collection are also time and resource-intensive (LeBlanc et al., 2016, Kahng et al., 2011, Zangrillo et al., 2021). Due to the critical nature of data collection and analysis, including how it may influence clinician and researcher decision-making, it is critical to develop solutions that increase the ease and efficiency of observational data collection.

There are several computer-based data collection solutions available that increase the ease at which researchers and clinicians perform observational data collection. Some of these software solutions are commercially available (Therapy Brands, 2022; Central Reach, 2022; ReThink Autism, 2022), but they may be financially unreasonable for researchers and clinicians, because they incur significant fees at either the organizational or user level. As an alternative, there is a history of clinician developed software solutions in Visual Basic and C++, some of which are publicly available (e.g., Dixon & MacLin, 2003; Bullock et al., 2017; Gilroy, 2017). Many of these softwares, such as *BDataPro* (Bullock et al., 2017), *BDataPro*’s beta version *DataPal 1.0* (Phipps et al., 2022), *PB.MI* (Zheng et al., 2022), *DataTracker* (Gilroy, 2017), and *PC_Eyewitness* (MacLin et al., 2005), have a focused set of features and functionality that align with present day needs but may present shortcomings for new experimental approaches. For instance, if a researcher wanted to code prerecorded video, then the options are limited to using either *DataPal 1.0* or *BDataPro* on one screen with the video open in another screen. In practice, this leads to misalignment of the video and behavioral data, which can be unacceptable in some studies.

As technology continues to become more accessible, it will likely lead to an increased number of behavior analytic clinicians employing body worn physiological sensors, video and audio recording, virtual reality, and machine learning into their practice and research (Arce & Gehringer, 2021a and b; Bone et al., 2016; Taylor & Lanovaz, 2022). For example, many of our studies incorporate either behavioral data with video data or behavioral data with physiological signals, such as those from wearable physiological sensors like the E4 by Empatica (hereafter referred to as Empatica E4). To our knowledge, there are no existing open-source data collection tools, designed for behavior analysts, that incorporate multiple data streams into a single interface. Thus, we decided to design a software package that meets our research needs.

The purpose of this article is to provide this software to researchers and clinicians free-of-charge, describe the functionality of the software program, *cometrics* (\ (ˌ)kō-ˈme-triks \), and provide documentation concerning its features and use. We designed *cometrics* to facilitate the transfer of data between a clinical environment and a research environment for the purposes of generating data that minimally intrudes on clinician ease of use.

## Supported Data Inputs

The *cometrics* software package supports the collection of three data sources:

1. behavioral or observational data using keystrokes (c.f., DataPal and BDataPro; Bullock et al., 2017);
2. physiological data from the Empatica E4; and
3. the synchronization of behavioral and physiological data with a video, either with or without audio.

To begin using *cometrics,* users must first create a Keystroke file. Keystroke files (KSFs) are structured spreadsheets that define the correspondence between a keystroke and a target behavior. Up to date and detailed information on the structure of KSFs can be found in the Appendix. For example, pressing key “a” to score an instance of hitting in a therapy session. In addition, the spreadsheet serves as an output file where patient-related sessions are stored. In the spreadsheet, we included a separate tab called “Conditions” where data collectors can separate specific conditions of assessment, treatment, or experiment (e.g., baseline, treatment, tangible, toy play). The conditions tab is used to populate a dropdown in the Session Window. When performing standard data analysis (e.g., calculating rate or proportion of session), Microsoft Excel cell referencing formulas can be used to aggregate dependent measures within the session. Raw and aggregate data are charted using the standard Microsoft Excel functionality. For those interested in generating APA quality graphs of observational data see Lehardy et al. (2021) and Mitteer et al. (2018). Users can generate a new revision of the KSF in the user interface, however, the formatting, equations, and sheets will be erased. Users would need to repopulate any cell referencing formulas in the document.

Due to our research interests involving the concurrent collection of physiological and observational data, we integrated Bluetooth support for the Empatica E4 sensor. The Empatica E4 is a wrist worn biosensor that collects four physiological measures (electrodermal activity, photoplethysmography, accelerometry, and temperature) from the subject. These data are streamed in real-time to the user interface using Empatica’s Streaming Server software package. To communicate with the Streaming Server, *cometrics* utilizes the pyEmpatica Python library developed by Arce & Gehringer, 2021a and b.

Video sources can either be a saved video or a real-time feed from a connected webcam. At startup, *cometrics* will detect any webcams connected to the user’s computer and present them as options in a dropdown menu in the “Video View” panel on the Session Window. Selecting a webcam input will start a real-time feed from that webcam and, when the session starts, *cometrics* will record the feed from that webcam into an MP4 file using the source resolution of the camera. In addition, if a microphone input is selected, that will be started simultaneously with the video feed and saved as part of the video.

## Getting Started

### Creating and Loading Projects and Patients

**Fig. 1:**
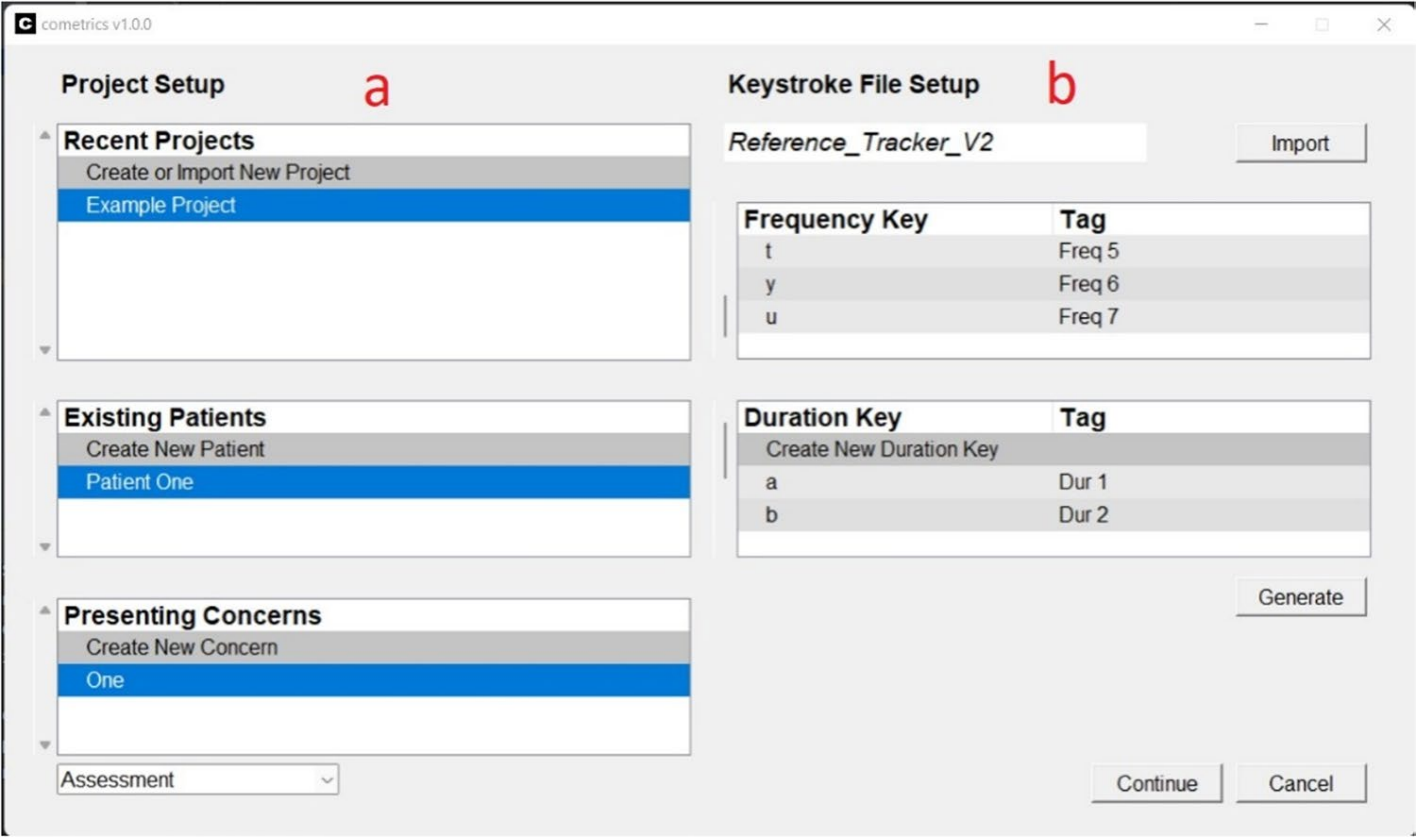
Project Setup

Upon launching the program, users are taken to the *cometrics* start menu (Fig. 1). The start menu is divided into two sections: Project Setup (Fig. 1, panel a) and Keystroke File Setup (Fig. 1, panel b). Project Setup is composed of the Recent Projects, Existing Patients, and Presenting Concerns sections. The Recent Projects tab is used to create new projects or import existing projects.

When users create a project, the root directory for the project is selected and a popup opens prompting for a project name using free-text entry. When the project name is selected, a folder under the root directory is created that houses all the data created for the project. When a project is loaded or created, the path to the directory is saved in a configuration file so that when *cometrics* is reopened, previous projects can be selected. Note that only the 20 most recent projects will be saved, and the oldest projects will be removed from the list.

Once a project is loaded, users can select or create a patient on the Existing Patients tab, again, users are prompted for a patient name. Once a patient is loaded users can select or create patient referral concerns. For example, if a patient is referred to for head-directed self-injury, a clinician may choose to indicate that as the referral concern. If the patient already has existing referral concerns, they will be loaded into the user interface. Concerns are populated in the same manner as the patient names.

When a concern is selected the phase type dropdown is enabled. Allowing for phase selection in the start menu allows for clinicians to better organize assessment and treatment data. The two phases enabled by default are Assessment and Treatment. Selecting one of these phases will create a folder with the name “{Concern} {Phase}” and subfolders: Graph, Raw Data, KSF, and Export. The Graph folder will store all graphed data including the populated KSF spreadsheet. The Export folder will store all exported data (e.g., sessions) in comma separated value format. The KSF folder will store all revisions of the KSF spreadsheet. The Raw Data folder will store all generated data separated by primary and reliability sessions.

Once the phase is selected, if the condition/phase folder exists, then the most recently created KSF will be loaded into the user interface (Fig. 1, panel b). If no file exists, a KSF file can be uploaded by selecting “Import” in the top righthand corner. The name of the uploaded KSF file will be viewable in the topmost input bar, next to the “Import” button. The Frequency Keys and Duration Keys are viewable in scroll views on the right side of the screen. Keys can be added to the KSF by double-clicking the first scroll view option labeled, “Create New Frequency/Duration Key.” When a new key is added, the “Generate” button will be enabled. When users click the generate button, *cometrics* will generate a new revision of the KSF. Pressing “Continue” will start the main Session Window.

### Beginning an Observation

**Fig. 2:**
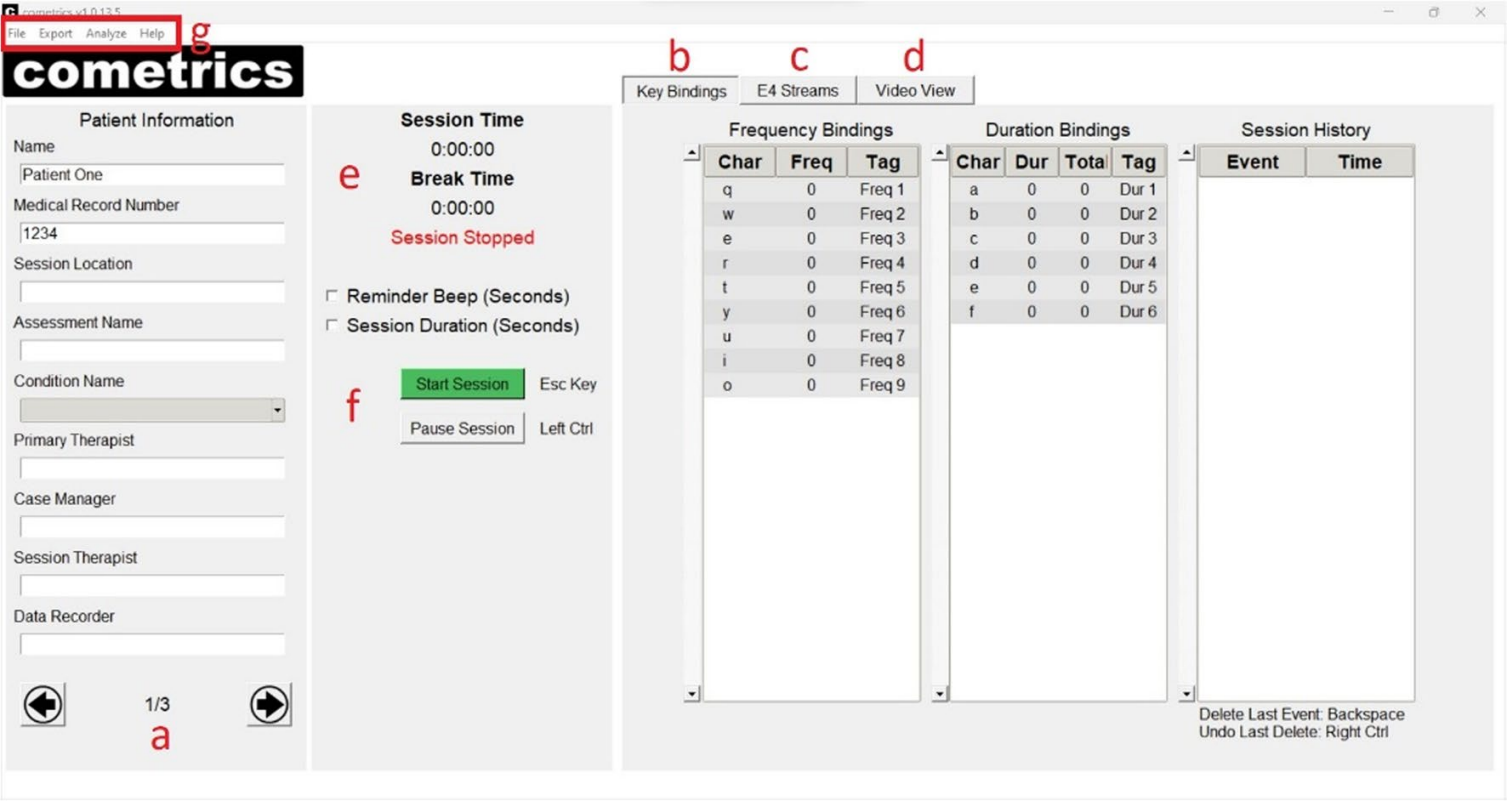
cometrics Main View and Key Bindings Tab

**Fig. 3:**
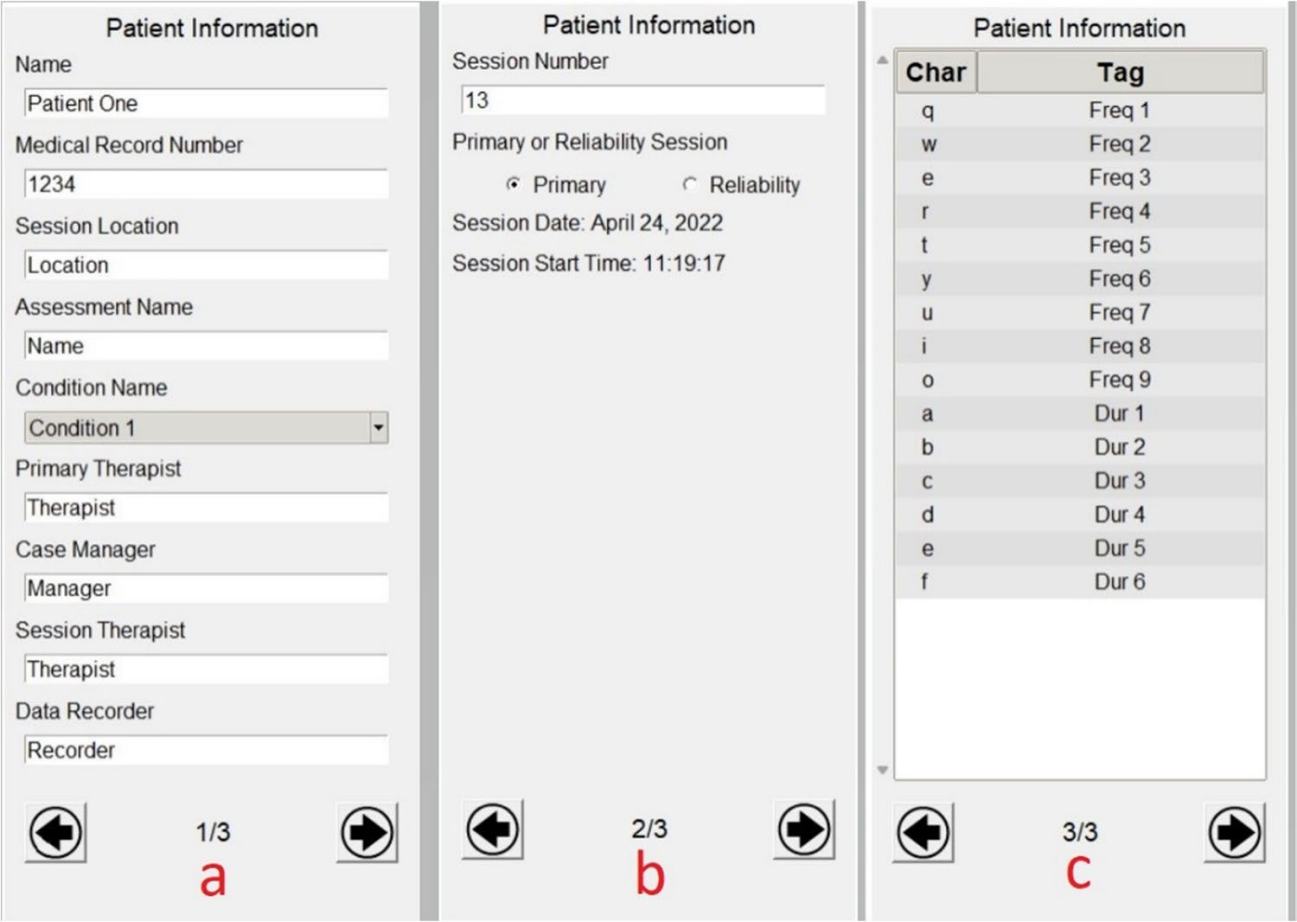
Patient Information Tabs

Once the project is set up and the user clicks “Continue,” the Session Window opens (Fig. 2), and session-specific information can be added. The left side of the Session Window (Fig. 2, panel a) provides entry fields for the session data. The Patient Information section (Fig. 3) consists of three pages, which are navigable using the arrows at the bottom of the section. The session cannot be started without filling out each field. The medical record number will be saved in the patient’s folder, which is loaded each time the project’s patient is loaded. Standard information such as the type of assessment, condition, therapists, and data recorder name can all be filled out on the first page of the Patient Information box (Fig. 3, panel a). Note that *cometrics* does resize the window based on the user’s screen size, so smaller screens will have fewer fields on the first page and some of the information shown will be moved to the second page. Setting the session to a primary or reliability session is performed on the second page of the Patient Information fields (Fig. 3, panel b). A primary session is the initial observation performed and the reliability session checks consistency of the measurement strategy. The second page of the Patient Information fields also shows the session number and the session start time. Session numbers can be changed using free-text input and session start time is automatically updated when the session starts. *Cometrics* checks if the session number chosen already exists in your collections, which will influence whether it is a primary or reliability session and prompts the user accordingly. To bypass this check, the session number must not be an integer, so if the user is collecting sessions based on the collection date or order of completion, as in the case of a retroactive data analysis or some other research study, the data files will have session identifying information attached. The final page contains the available keystrokes, allowing the user to see available keystrokes in another view during the session (Fig. 3, panel c).

**Fig. 4:**
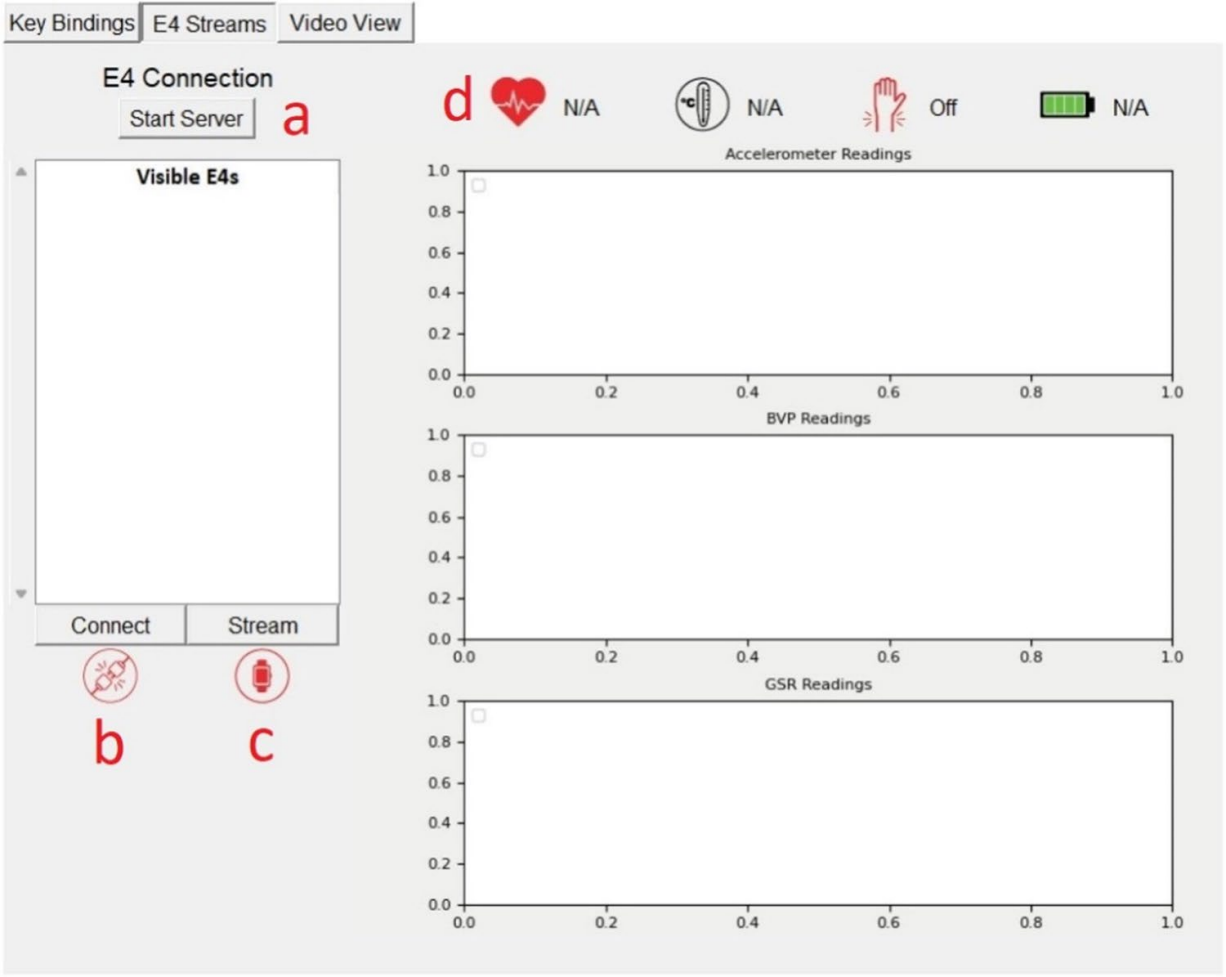
E4 Streams Tab

On the right side of the Session Window, there are three data panels: Key Bindings (Fig. 2, panel b), E4 Streams (Fig. 2, panel c), and Video View (Fig. 2, panel d). These tabs can be viewed by clicking on their headers. The Key Bindings panel provides a listing of the assigned keystrokes and the session history. When the session starts, the detected keystrokes are listed along with the second at which they occurred in the session. The E4 Streams panel provides the interface required to find and connect to Empatica E4 devices on the E4 Streaming Server provides a real-time graphic display of the Empatica E4 data streams. To connect an Empatica E4 to *cometrics* the E4 Streaming Server, provided by Empatica, must be running and the user’s Empatica E4 must be connected to it. Once connected to the server, pressing the “Start Server” button (Fig. 4, panel a) will connect *cometrics* to the E4 Streaming Server and the text in the button will change to “List Devices.” Upon clicking “List Devices,” the devices connected to the E4 Streaming Server are populated. The user then selects the device from the “Visible E4s” list and, once highlighted, presses the “Connect” button (Fig. 4, panel b). The graphic under the button will change to a green icon, indicating that *cometrics* is able to communicate with the Empatica E4 and the button’s text will change to “Disconnect.” Clicking the “Stream” button (Fig. 4, panel c) will start the data streams, which will automatically display on the right side of the panel (Fig. 4, panel d). The graphic under the “Stream” button will change to a green icon indicating successful streaming connection. To disconnect from the Empatica E4, either end the session or press the “Disconnect” button.

The middle panel is the session time tracker (Fig. 2, panel e), which maintains a timer showing the current session time and the current pause time. The top checkbox is used as an interval timer, which allows users to utilize discontinuous measurement strategies, where intervals are indicated with an audible beep. The bottom checkbox caps the session at a prespecified duration. Upon reaching that time limit, *cometrics* will end the session automatically. The “Start Session” button will begin the session providing all Patient Information is complete (Fig. 2, panel f). As an alternative, the Escape key (Esc) can be pressed to start the session. The “Pause Session” button pauses the session timer and starts the break timer. As an alternative, the Left Control (Ctrl) button can be pressed to pause the session. To resume the session, press the Left Control (Ctrl) button again.

**Fig. 5:**
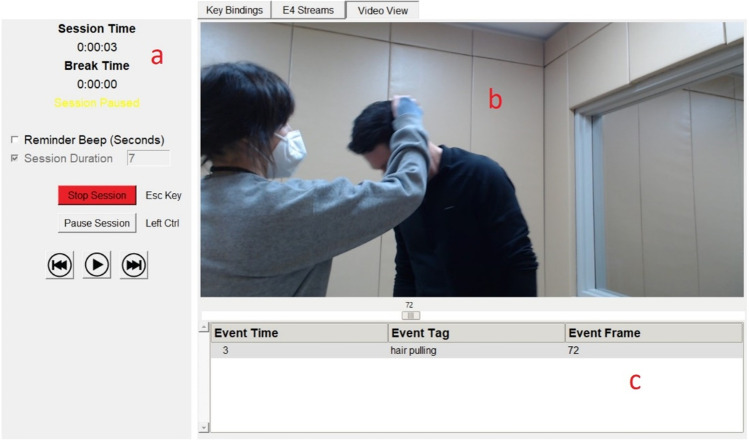
Video View Panel

**Fig. 6:**
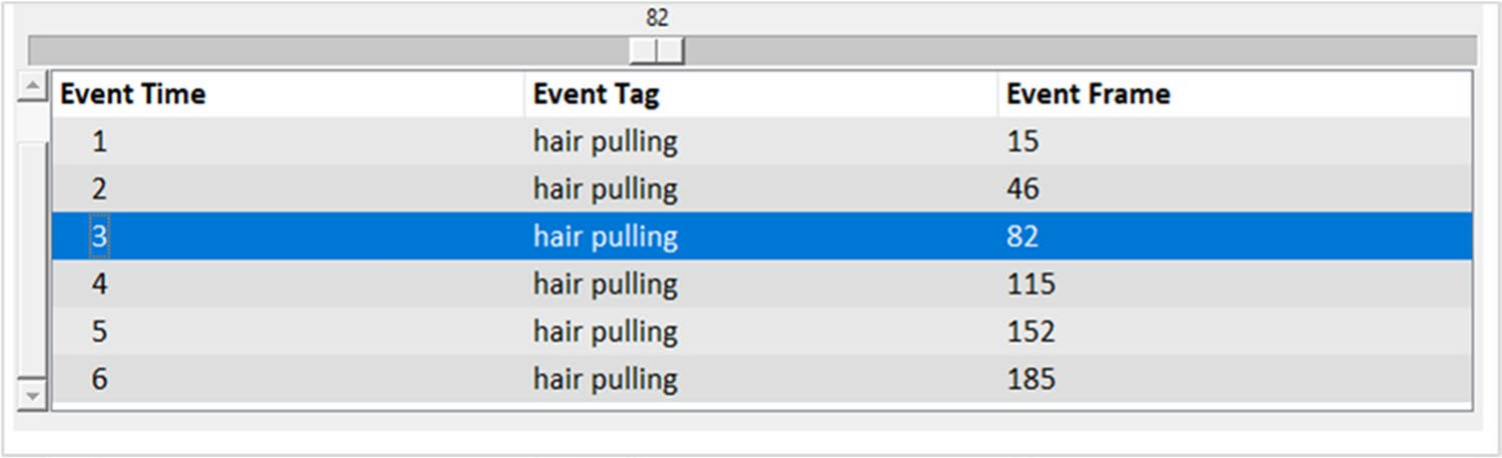
Session Event Viewer

As mentioned above, users have the option to load pre-recorded video in the Video View panel (Fig. 5, panel a). Loading a video will automatically populate video control buttons. The play button will start the session and the seek buttons on either side of the play button will skip the video forward and backward by one second (Fig. 5, panel c). When using prerecorded videos, session duration is automatically set to the length of the video. When a session is started, the loaded video will begin playing and recorded events are documented in the event viewer below the video (Fig. 5, panel b). The event viewer shows the specific event tag, the timeframe in which the event occurred, and the exact video frame at which the event was tagged (Fig. 6). If events are recorded in error, the most recent event can be deleted by pressing the Backspace button. If an event is deleted in error, users can restore the event by pressing the Right Control (Ctrl) button.

When the session ends, either manually or automatically, the session data is saved to the selected output directory along with any other complimentary data such as a copy of the video, the recorded video, or the Empatica E4 data (if applicable). When the data successfully saves, a popup box will ask the user if they want the File Explorer to open the output file location, allowing for data verification.

Saved data can be compiled into two formats, either a sequential list of sessions using the Keystroke File spreadsheet or as separate CSV files. When the Keystroke file is populated, each session is listed in numeric order starting from session 1 with each keystroke bin populated with the number of recorded instances of that keystroke in the session. As an alternative, when the CSV files are populated, the session information is populated at the top of the CSV file and each individual keystroke is listed in sequential order. These functions are accessible under the “Analyze” and “Export” tab in the menu bar at the top of the screen, respectively (Fig. 2, panel g).

### Calculating Interobserver Agreement

**Fig. 7:**
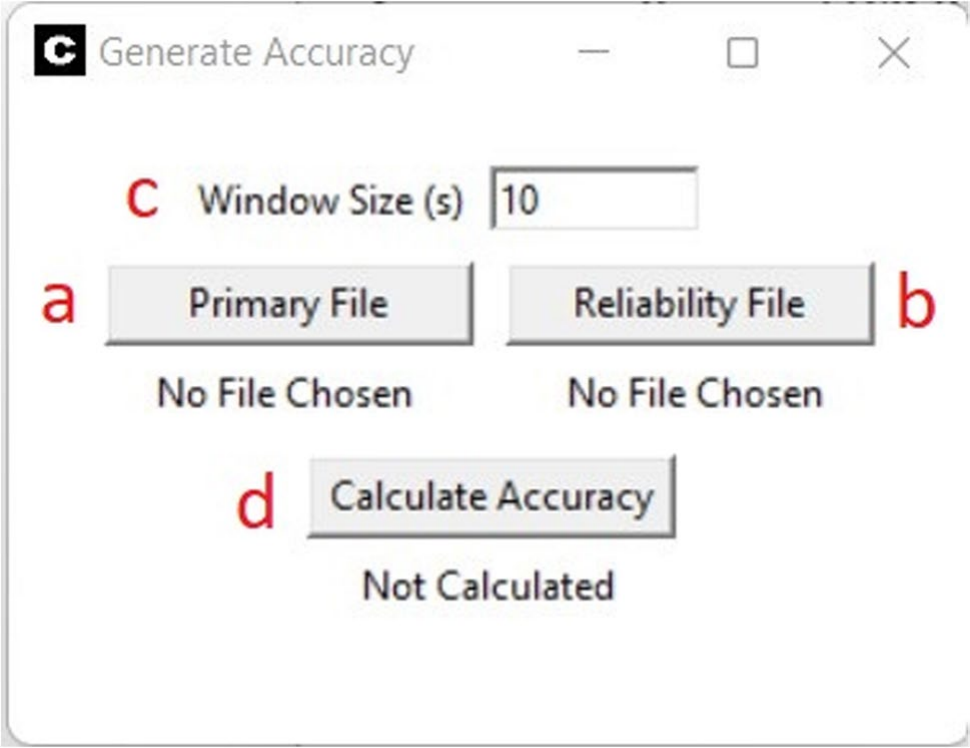
Interobserver Agreement Setup Window

To calculate interobserver agreement, users must select the “Calculate Session Accuracy” button, found under the “Analyze” Menu option (Fig. 2, panel g). The popup that generates will prompt for two files: the primary session file and the reliability session file (Fig. 7, panel a and b). In addition, the window size of the analysis can be modified depending on the needs of the clinician or researcher (Fig. 7, panel c).

Pressing the “Calculate Accuracy” (Fig. 7, panel d) button will generate a Microsoft Excel spreadsheet that includes individual tabs listing the raw primary data, the raw reliability data, and the calculated agreement coefficients separated by the specified window size.

## Data Organization and Usage

*Cometrics* is designed to organize projects under a root project directory. Each project has a subfolder that has the same name as the project. Inside each project folder, a subfolder named after the patient exists for each patient folder. Within each patient folder is a concern file, patient file, and subfolders for each concern phase. The concern phase folders contain a standard directory that consists of four folders by default: Export, Graph, KSF, and Raw Data. These folders are where data is stored by the software.

Data generated in *cometrics* can be exported into multiple formats directly from the user interface. If the session has an associated video, a copy of the video will be stored in the Raw Data folder with the same name as the session. Session files will be named in the following format:

{session number} {first two letters of the assessment} {first two letters of the condition name}{session date (DDMYYYY)}{“_R” if a reliability session}

Raw session data are stored using the JSON file format, making it readily interpretable by software for advanced analysis of session data while remaining human readable. For instance, reading the session output files into a Python script would only require the use of the built in JSON library whereas to read the file into an R script would only require the use of the rjson library. There are multiple fields within the session file and session specific information, such as keystroke files and the values from the Patient Information fields (Fig. 3), are copied so no other files are needed for processing.

Applications of *cometrics* at a local level have resulted in student led poster presentations, where Kramer et al. (2022) used simultaneously recorded physiological data and observational data were used to determine heart rate differences between their coded conditions using v1.2.7 of *cometrics*. In addition, Davies et al. (2022) detailed an approach taken to code 160 hr of clinical recordings for challenging behaviors during the 2022 Summer semester using v1.1.4 of *cometrics* for use in a machine learning study.

## Menu Bar Navigation

The menu bar (Fig. 2, panel g) provides an interface to perform common actions, such as restarting *cometrics*, opening a new project, opening the user guide, or submitting feedback tickets. Full detail for the action each button performs can be found in the *cometrics* user guide and a brief description of each button is given in Table 1.

**Table 1:** Menu Bar Option Description

Menu Option	Submenu Options	Description
File	Start New Session Open New Project Connect External Input	Reset the coding UI with the same settings Close the coding UI and restart cometrics External devices can be added via Bluetooth for use during coding. Connected devices (clicker, mouse, etc.) can be used as an external button
	Edit Config File	Opens a popup user interface that allows the modification of persistent variables
Export	Export CSV	Used to export all existing session data for the patient into CSV files
Analyze	Analyze Sessions	Plots the session history for the patient into their KSF
	Calculate Session Accuracy	Calculate the interobserver metrics between two sessions
	Calculate E4 Metrics	Calculate statistical features of E4 data streams in previous sessions for patient for photoplethysmography and electrodermal activity.
Help	Open Documentation	Opens the user guide using default PDF viewer
	Open Logs	Opens the log file directory using File Explorer
	Open Current Directory	Opens the working directory for the current patient
	Download E4 Streaming Server	Opens the documentation for setting up the E4 Streaming Server and automatically downloads the same
	Open Source Code	Opens the default web browser to the public GitHub page for the cometrics software package
	View Privacy Policy	Opens cometrics privacy policy using the default browser
	Submit Feedback	Opens popup that collects feedback and sends it to the developer. This feedback will be posted as an Issue on the GitHub repository

## Validation and Testing

To validate this new software package, we performed a series of tests similar to the validation testing done for the *BDataPro* software package (Bullock et al., 2017). *Cometrics* was tested to ensure that (1) the timing is accurate and steady; (2) the recorded events (behavioral, physiological, video, and audio) are accurate and reliable; (3) the summary statistics are accurate and reliable; (4) the reliability coefficient calculations are accurate and reliable; and (5) that the software operates across multiple makes and models of personal computers using Windows 10 and 11. This testing can also be reconfirmed by the user for accuracy by following the procedure proposed for *BDataPro* in Appendix B of Bullock et al.

## Bug Reporting

**Fig. 8:**
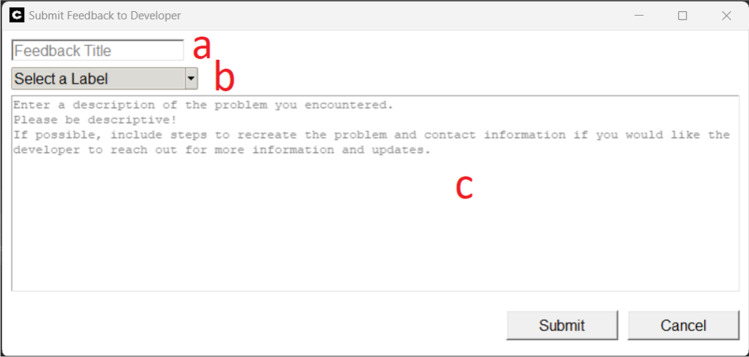
Bug Reporting User Interface

No software is perfect, and problems may arise that impede the user’s ability to interact with *cometrics*. To resolve the address any unforeseen issues, we created a method to report feedback and issues anonymously to the maintainers of *cometrics*. This process can be found in Section 24 of the *cometrics* user guide. To submit a ticket, a title needs to be provided (Fig. 8, panel a), a label of either bug, enhancement, or documentation needs to be assigned (Fig. 8, panel b), and a description for the feedback needs to be provided (Fig. 8, panel c). Although the feedback is anonymous, it may be useful to include a contact method, such as an email address, so clarifying questions may be asked, and user problems can be resolved quickly.

For more complex issues, the maintainers may ask for the log file associated with your bug, if contact information is provided. To find your log files, use the menu tab option “Open Logs” (Table 1, column 11).

## Availability of *cometrics*

To encourage collaboration and community support, *cometrics* is a free and open-source software (FOSS) distributed under an MIT license, with the Python 3.8 source code available on GitHub. An MIT license is a short and simple license for software which only requires that the copyright and license be preserved when modifying and using the software. The MIT FOSS license allows *cometrics* to be modified, used, and distributed freely if the license and copyright are preserved in the software. To improve access to *cometrics* we have listed it on the Microsoft Store, making it less likely to be blocked by your institution’s firewall and allowing for automatic updates. On GitHub, compiled binaries can be found that have been tested on Windows 10 and 11.

An additional benefit of open-source software is that it aligns with a dimension of applied behavior analysis outlined in Baer et al. (1968). The dimension of *Technological* specifies that a method or procedure used is clearly described to facilitate application and replication. Developing software under open-source licenses allows any interested party to examine the source code, contribute to the development of the software, and modify the existing software to meet their specific needs. Further benefits of publishing software under open source licenses includes the ability of knowledgeable users to investigate any issues they may have with the software. For example, if users encounter a software error, they can investigate the line(s) of code responsible for the error and resolve that issue on their own by modifying the code and submitting the fixed code to the GitHub repository, to share this fix with the community (Gilroy & Kaplan, 2019). In addition, open source software allows users to perform their own validation of the software. Users can observe and interact with each line of code to see exactly how the software takes converts user input to the software outputs discussed in this article.

## Conclusion

We developed *cometrics* as part of a collaboration between the Munroe-Meyer Institute’s Severe Behavior Department at the University of Nebraska Medical Center and Munroe Meyer Institute’s Virtual Reality Laboratory at the University of Nebraska Medical Center. With the aim of facilitating current and future research endeavors, we focused on bridging a technological gap in observational data recording by synchronizing multiple sources of data on a multimedia timeline. By making *cometrics* available on the Microsoft Store, the barrier to entry for clinicians to access and use the software is dramatically decreased. We consider *cometrics* a living project and so there will be bug fixes and feature additions as we work with the community that may not be reflected in this document. We strongly encourage readers to reference the *cometrics* user manual for the most current information regarding features and functionality.

### Supplementary Information

ESM 1 (XLSX 9 kb)

## Data Availability

All data generated and used for this current study are publicly available in the Virtual Reality Laboratory’s *cometrics* repository, https://github.com/Munroe-Meyer-Institute-VR-Laboratory/cometrics.

## Appendix

For full information on expected input and output files and the required formatting of Keystroke Files, read the following tutorial: https://github.com/Munroe-Meyer-Institute-VR-Laboratory/cometrics/blob/main/tutorials/example_output_ksf_intro/example_output_ksf_intro_tutorial.md
